# Timeliness and quality of peripartum care provision during a health system strengthening initiative in rural Guinea-Bissau: a qualitative situation analysis

**DOI:** 10.1186/s12884-024-06669-8

**Published:** 2024-07-13

**Authors:** Sabine Margarete Damerow, Helene Vernon Adrian, Bucar Indjai, Elsi José Carlos Cá, Nanna Maaløe, Ane Bærent Fisker, Jane Brandt Sørensen

**Affiliations:** 1 Bandim Health Project, Research Unit OPEN, Department of Clinical Research, University of Southern Denmark, Studiestræde 6, Copenhagen K, 1455 Denmark; 2 Bandim Health Project, INDEPTH Network, Bissau, Guinea-Bissau; 3 National Institute for Studies and Research (INEP), Bissau, Guinea-Bissau; 4 Global Health Section, Department of Public Health, University of Copenhagen, Copenhagen, Denmark; 5 Department of Gynaecology and Obstetrics, Copenhagen University Hospital – Herlev Hospital, Copenhagen, Denmark

**Keywords:** Maternal and child health, Health systems strengthening, Systems thinking, Quality of care, Universal health coverage, Guinea-Bissau

## Abstract

Guinea-Bissau has among the world’s highest maternal and perinatal mortality rates. To improve access to quality maternal and child health (MCH) services and thereby reduce mortality, a national health system strengthening initiative has been implemented. However, despite improved coverage of MCH services, perinatal mortality remained high. Using a systems-thinking lens, we conducted a situation analysis to explore factors shaping timeliness and quality of facility-based care during labour, childbirth, and the immediate postpartum period in rural Guinea-Bissau. We implemented in-depth interviews with eight peripartum care providers and participant observations at two health facilities (192 h) in 2021-22, and analysed interview transcripts and field notes using thematic network analysis. While providers considered health facilities as the only reasonable place of birth and promoted facility birth uptake, timeliness and quality of care were severely compromised by geographical, material and human-resource constraints. Providers especially experienced a lack of human resources and materials (e.g., essential medicines, consumables, appropriate equipment), and explained material constraints by discontinued donor supplies. In response, providers applied several adaptation strategies including prescribing materials for private purchase, omitting tests, and delegating tasks to birth companions. Consequences included financial barriers to care, compromised patient and occupational safety, delays, and diffusion of health worker responsibilities. Further, providers explained that in response to persisting access barriers, women conditioned care seeking on their perceived risk of developing birthing complications. Our findings highlight the need for continuous monitoring of factors constraining timeliness and quality of essential MCH services during the implementation of health system strengthening initiatives.

**Supplementary Information:**

The online version contains supplementary material available at 10.1186/s12884-024-06669-8.

## Introduction

While considerable global progress in improving maternal and perinatal survival has been made, sub-Saharan Africa is lagging behind [1, 2]. With most recent estimates of 536 maternal deaths per 100,000 live births, 21 stillbirths per 1,000 births, and 27 neonatal deaths per 1,000 live births [1], sub-Saharan Africa is the world region farthest from attaining the global 2030 targets [3–5]. In the region, high mortality coincides with suboptimal coverage of essential maternal and child health (MCH) services: In 2015-21, coverage of skilled birth attendance was estimated at 64% [6]; coverage of four or more antenatal care (ANC) consultations (ANC4) below 54% [7]; and even upon attaining care by skilled attendants, quality of ANC and intrapartum care often remains suboptimal.

Strong health systems are considered the premise for universal access to quality MCH services [8]. Consequently, health systems strengthening has gained increased attention as an approach to increase coverage and quality of MCH services and reduce maternal and perinatal mortality. Health systems strengthening is an umbrella term for systemic improvements of health system core functions (leadership/governance, financing, workforce, medical products, technologies, information and research, service delivery) and their interactions aiming at attaining sustainable improvements of health services and health outcomes [8]. Yet, the effects of the health systems strengthening initiatives for MCH implemented in various sub-Saharan African settings are mixed, and many initiatives have failed to achieve improved MCH outcomes [9–13], sometimes despite concurrent increases in MCH service uptake [9, 10, 13].

Here, three factors may be decisive: First, health systems are greatly context specific [8]. Hence, to improve MCH outcomes, the identification of the system-specific constraints determining the inadequate MCH outcomes is fundamental to conceiving adequate countermeasures. Meanwhile, failure in the adequate contextualisation of health systems strengthening initiatives likely undermines their effectiveness. Second, system-level interventions are typically complex in nature, and interact with commonly complex implementation contexts [14]. Therefore, even if the “right” constraints and solutions are identified, the effects of health systems strengthening initiatives are hard to predict. Hence, at times, seemingly adequate solutions fail to achieve anticipated effects [14]. Third, implementation contexts are not only commonly complex, but also dynamic and may change over time [14]. For example, low-income countries are often highly dependent on donor support for health financing, with external funding frequently exceeding government contributions [15]. At the same time, external country support for MCH is often highly volatile [16], thereby constantly changing recipient countries’ health financing frameworks and the premises to secure and budget adequate funds to sustain implemented interventions. However, despite the growing popularity of health systems strengthening initiatives, mechanisms determining successes and failures of implemented initiatives remain understudied. This undermines the generation of best practices for the design and implementation of effective initiatives.

Guinea-Bissau is a low-income country in West Africa with among the world’s highest maternal and perinatal mortality rates [1]. Here, the ‘Integrated Programme for the Reduction of Maternal and Child Mortality’ (PIMI) has been implemented, a national health system strengthening initiative aiming at improving maternal and child survival through better access to quality MCH services [17–19]. PIMI comprises a comprehensive intervention package including user-fee waivers, health worker trainings, supplies of medical equipment, medicines and consumables, facility maintenance and rehabilitation, and subsidisation of ambulance services [17–19]. However, PIMI’s impact has fallen behind expectations: while population coverage of ANC4 and facility births increased markedly during PIMI’s implementation in rural Guinea-Bissau, from approximately 1/3 pregnancies pre-PIMI to 1/2 during the initiative’s nationwide implementation, coverage remained far from universal [20]. Moreover, coverage increases were similar between PIMI’s early and late implementation areas, i.e., areas where the initiative was first piloted vs. areas where it was only introduced during its nation-wide roll-out, suggesting that improvements were likely secular rather than associated with PIMI [20]. At the same time, despite overall increases in ANC4 and facility birth coverage, perinatal mortality (stillbirths and neonatal deaths < 7 days) remained high and stable at around 80 deaths per 1,000 births, suggesting that quality of MCH services remained suboptimal [20].

Our previous demand-side assessment of factors compromising women’s access to facility births in rural Guinea-Bissau identified ubiquitous out-of-pocket payments (OOPs) alongside geographical barriers despite PIMI’s implementation [21]. Hence, persisting access barriers likely explain why coverage developments have fallen behind expectations during PIMI. However, it has not yet been explored why general coverage increases did not translate into improved perinatal survival during PIMI. Against this background and with a view to contribute to a better understanding of mechanisms determining the effects of health systems strengthening initiatives, the aim of this study was to explore factors shaping the provision of timely and quality peripartum care during PIMI in rural Guinea-Bissau.

## Materials and methods

### Study setting

Guinea-Bissau is considered one of the world’s poorest and politically most unstable countries [22]. Here, access to health services is severely compromised: The health system is underfunded and OOPs are the key health financing source, estimated at 64% of the current health expenditure [23]. Meanwhile, poor road conditions and inadequate transportation networks compound physical accessibility of health services, and health facility conditions are often substandard with frequently lacking electricity, water supplies, essential medicines and equipment [24]. Moreover, there is a severe health worker shortage [24]; the country’s physician density is estimated at just 2 per 10,000 people [25].

PIMI was initiated with a focus on strengthening access to public MCH services and their quality for all pregnant and birthing women up to 45 days postpartum and children below five years of age. First, PIMI was implemented as a pilot in four of Guinea-Bissau’s ten rural health regions from 2013 to 2016. Since 2017, PIMI has been rolled out to all public health facilities across the country [17, 19, 26], except for the National Hospital Simão Mendes where Médecins Sans Frontières had implemented another MCH initiative. Hence, in usual absence of private providers in the rural areas, PIMI has been shaping the MCH service delivery landscape in rural Guinea-Bissau since its implementation.

With insufficient domestic funds, Guinea-Bissau is highly dependent on donor support to enable the provision of essential health services [23]. Hence, to date, PIMI has been supported through donor funding: From 2013 to 2021, through funding from the European Union (EU) with smaller contributions from UNICEF and international non-governmental organisations (NGOs) who also served as implementation partners [17–19]. In July 2021, EU funding ceased and PIMI’s core activities were transferred to the World Bank’s country health programme [27]. Recently, in June 2022, the EU entered again as PIMI’s core funder. By mid-2025, PIMI is planned to be transitioned to domestic financing [26]. In this study, we refer to PIMI as the implemented health system strengthening initiative independent of the current financing partner.

In Guinea-Bissau, public MCH services are organised into three levels [28]. Health centres usually provide decentralised primary health services in smaller towns, including ANC and intrapartum care for uncomplicated childbirths. Regional hospitals and large health centres provide more comprehensive birth assistance in larger towns, usually including basic surgical procedures. The National Hospital Simão Mendes provides centralised tertiary care in the capital Bissau. During ANCs, triage is performed, and women categorised as being at high risk of developing birthing complications are referred to regional hospitals or the national hospital for intrapartum care; women categorised as being at low risk remain at their target health facility, usually a health centre. Women developing birthing complications exceeding the care standard of a health facility are commonly referred to the appropriate higher care level, which often requires journey times of several hours over land and/or water.

### Study design

We conducted a situation analysis to explore factors shaping the timeliness and quality of peripartum care provision during PIMI in rural Guinea-Bissau, nested in a wider project evaluating PIMI’s effects on MCH service coverage and perinatal mortality [20], and barriers to care [21]. To capitalise on contextual insights gained during preceding evaluation components, we conducted this study at the target health facilities of women who previously participated in household-based in-depth interviews (IDIs) on birthing experiences and barriers to facility births [21].

For the present study, we took a supply-side perspective and explored provider perceptions of the status quo of peripartum care provision and challenges, as well as actual practices at the point of care. We defined peripartum care as care provided to birthing women during health facility admission, including care during labour, childbirth, and the immediate postpartum period until health facility discharge. The selected study sites were located in four health regions. They included three public health centres, three public regional hospitals, and two NGO-operated maternity waiting homes adjacent to two of the regional hospitals, where women with high-risk pregnancies could stay in the time leading up to the birth (Table 1). We conducted IDIs (*n* = 8) with each health facility’s responsible peripartum care provider and the persons in charge of the maternity waiting homes (i.e., non-medical personnel trained in community health and health education), thereby following a purposive key informant strategy (i.e., a selection of interviewees based on their knowledge of peripartum care provision and challenges in rural Guinea-Bissau) [29]. If more than one responsible peripartum care provider was available at a health facility, we asked the most senior provider for their participation. To gain in-depth insights into the actual provision of timely and quality peripartum care, we complemented IDIs by 192 h direct observations at the regional hospital and health centre in one region (Table 1).

**Table 1:** Study sites, performed data collections, and in-depth interview participants

	Data collection	Study sites	In-depth interview participants^1^
**Region 1**	In-depth interviews and direct observations	Health centre Regional hospital	Head nurse of the health centre Head midwife of the maternity ward
**Region 2**	In-depth interviews	Health centre Regional hospital Maternity waiting home	Nurse for maternal and child health services Head midwife of the maternity ward Person in charge of the maternity waiting home
**Region 3** ^2^	In-depth interviews	Regional hospital Maternity waiting home	Head midwife of the maternity ward Person in charge of the maternity waiting home
**Region 4**	In-depth interviews	Health centre	Midwife responsible for reproductive health services in the health area

^1^ Personal details are omitted to ensure participants’ anonymity

^2^ No health centre was included in region 3 since the target health centre was inaccessible when we conducted the in-depth interviews

### Conceptual framework

We used a systems-thinking lens [14, 30] to explore factors shaping the provision of timely and quality peripartum care. Thereby, we acknowledged that peripartum care is embedded in complex interactions between health system characteristics, PIMI’s policy package, clinical processes, institutions, individual service providers, women, and contextual factors. This likely influences timeliness and quality of care. Using a systems-thinking lens further motivated us to focus our attention on (i) underlying system dynamics impacting timeliness and quality of peripartum care and (ii) providers’ and women’s adaptation strategies to factors compromising timeliness and quality of peripartum care. Here, we distinguished “responses” from “tactics”: If an adaptation strategy appears to be the only possible course of action, we refer to it as a “response”; if actors are perceived to be able to choose between alternative courses of action, we refer to it as a “tactic”.

**Fig. 1:**
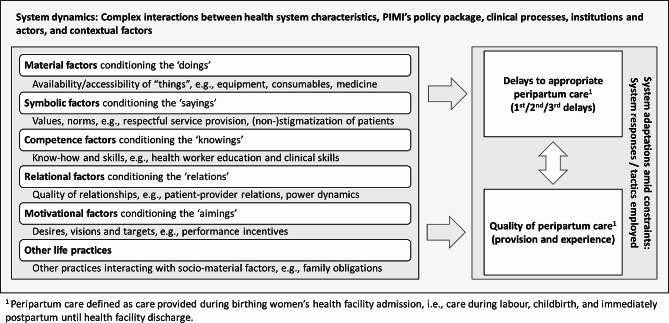
Conceptual framework of the study drawing on the social practice framework developed by Skovdal [32]

Further, taking inspiration from contemporary theories of social practice [31, 32], we approached peripartum care provision as a social practice. To examine factors promoting or compromising timeliness and quality of peripartum care, we employed the social practice framework developed by Skovdal [32]. Thus, we categorised factors as *material* (availability of “things”, e.g., equipment, medicine), *symbolic* (values, norms, e.g., respectful service provision), *competence* (know-how and skills, e.g., health worker education), *relational* (quality of relationships, e.g., patient-provider relations), and *motivational* (desires, visions and targets, e.g., performance incentives), while also drawing attention to *other life practices* (e.g., family obligations). Aided by the ‘Three Delays Model’ [33], we further distinguished compromised timeliness of care across time and space into delays associated with the decision-making of seeking care (1st delay), reaching the health facility (2nd delay), and obtaining care at the health facility (3rd delay). Further, aided by the World Health Organization (WHO) framework for quality maternal and newborn care [34], we considered care provision and experience as linked dimensions of quality of care. Figure 1 provides a summary of the study’s conceptual framework.

### Data collection

IDIs were conducted in September and October 2021, supported by an interview guide focussing on provided peripartum services and challenges to the provision of adequate care (cf. Supplementary Material). The interviews were conducted in Guinea-Bissau Creole by author 3 (male, Bissau-Guinean) in collaboration with author 1 (female, European, stationed in Guinea-Bissau, speaks Guinea-Bissau Creole). Interviews were audio recorded and verbatim transcribed in Guinea-Bissau Creole. They were further complemented by field notes detailing, e.g., the interview setting and non-verbal impressions. Member checking was applied by summarising elicited key information at the end of each IDI and asking whether the participant deemed this accurate (cf. Supplementary Material).

Direct observations were conducted during a four-week period (health centre: 2.5 weeks; regional hospital: 1.5 weeks) at differing times during day and night in March and April 2022. We focussed the observations on physical settings, clinical practices, and interactions between providers, women and birth companions during labour and birth, aided by an observation guide (cf. Supplementary Material). Waiting areas, ANC and postnatal care were also observed (cf. Supplementary Material). Observations were conducted with moderate participation, i.e., during observations, we engaged in informal conversations with providers and women and supported providers by e.g., handing equipment, but we did not engage in clinical tasks. Observations were conducted by author 2 (female, European, registered nurse, stationed in Guinea-Bissau, speaks Guinea-Bissau Creole) in collaboration with author 1, and documented in field notes.

### Data analyses

We applied thematic network analysis [35] in an inductive-deductive approach guided by our conceptional framework. First, we inductively generated basic themes of factors shaping the provision of timely and quality peripartum care. Second, we organised the basic themes into thematic networks. To this end, we deductively classified all basic themes into predefined middle-order organising themes (material, symbolic, competence, relational, and motivational factors, and other life practices) and inductively generated four superordinate global themes which are grouping similar basic themes (promotion of facility-based peripartum care, geographical, material, and human-resource constraints). The structure of our reporting of findings is guided by the global themes while constraints are reported in the sequence of resulting delays to care. Coding was conducted by author 1 based on verbatim transcripts, field and observation notes using NVivo 12 Pro / NVivo Enterprise Pro 2020 [36, 37]. Generated themes were discussed within the study team and preliminary findings in two workshops with peripartum care providers from both observation sites in August 2023 to determine whether they perceived the findings to reflect their realities. Reported quotes were extracted from the verbatim transcripts, translated to English by author 1 and checked for content accuracy by author 6. All reported payments were recorded in West African CFA Franc (XOF) and converted into 2022 United States Dollar (USD) using the official period average exchange rates published by the World Bank [38], adjusted for inflation using the United States consumer price index rates derived from the International Monetary Fund [39]. For payments in 2021, values were converted at 1 USD = 554.53 XOF [38] and adjusted for 8% inflation [39]; for payments in 2022, values were converted at 1 USD = 623.76 XOF [38].

### Ethics

Ethical approval was granted by the National Ethics Committee of Guinea-Bissau (122/CNES/INASA/2020, 020/CNES/INASA/2021, and 018/CNES/INASA/2022); administrative approval granting access to the study sites by the regional health authorities and the participating health facilities. Written informed consent was obtained from interviewed and directly observed providers. We carefully ensured that IDIs and observations did not interfere with providers’ clinical obligations. In collaboration with attending providers, women and companions who were present during our observations were orally informed about the study. Women’s oral consent was obtained for observations of situations warranting privacy, such as medical history interviews, physical examinations, or births. Here, we opted for oral consent since a written process was not feasible due to the risk of delaying care obtainment and interfering with clinical priorities. To ensure anonymity and confidentiality, we masked all identifiable personal and health facility information during data analyses and reporting.

## Findings

### Promotion of facility-based peripartum care

In IDIs and observations, providers stressed that health facilities were the only reasonable place to give birth since home births were associated with elevated risks, especially when complications arose:

“At home, you don’t know the foetal presentation, and that can cause them to come here with complications, for example haemorrhage and many other things … Though a home birth can go well, there is still the risk of haemorrhage after birth, and the way they cut the child’s umbilical cord can cause infections… if you don’t notice this, this can kill the child.” (Head midwife, regional hospital, region 1).

To promote peripartum care uptake, interviewees commonly emphasised that health education, provided during ANC and ideally complemented by outreach measures targeting more remote villages, was key:

“They come to the health facility [for childbirth], often they come, that’s because of the counselling.” (Head midwife, regional hospital, region 2).

Interviewees also stressed the importance of involving women’s key relations, i.e., partners, families, and ideally the whole community, into health education to ensure it was not later overruled by conflicting opinions or traditions. Furthermore, they highlighted the importance of providing a good care experience and reputation and explained that user-fee waivers covering service provision, medicines and consumables were key enablers to promote care uptake:

“Everything is free of charge here – when you give birth here you don’t even pay 25 francs [coin with the lowest value], you pay nothing.” (Head midwife, regional hospital, region 3).

Yet, during observations, we noted that ANC consultations were usually very short and characterised by limited communication between providers and women. In this context, we noted that women were commonly not informed about why prescribed medicine should be taken, why examinations and tests were performed, the actual performance of procedures, and their results. Likewise, women were rarely invited to ask questions and engage in clinical decision making. We also observed diverse instances of harsh interactions between providers and women, including women being scolded for behaviour non-compliant with provided instructions, e.g., not purchasing all prescribed medicine, undressing too slowly, and not seeking all recommended ANC consultations. During ANC consultations, women were also seldomly accompanied by partners or family members. This stood in contrast to births, where female family members usually stayed at the health facility to support the birthing woman throughout her admission, e.g., with providing food and water. Further, despite formal user-fee waivers, we observed a multitude of direct and indirect OOPs required for the obtainment of ANC and peripartum care alongside other barriers to timely and quality care.

### Geographical constraints compromising uptake and timeliness of care

Most interviewees highlighted geographical hurdles as a key barrier to timely peripartum care provision. This was due to health facilities’ big catchment areas along with bad roads:

“It can take over three hours [to reach the hospital] … villages are far away, and, in parts of this region, the roads are bad, it’s taking a lot of time.” (Head midwife, regional hospital, region 2).

Long travel times were reported to coincide with difficulties in accessing means of transportation. The study sites had ambulances which could be called upon onset of labour, usually one or two vehicles per health facility serving for all medical emergencies including referrals. However, ambulance transport was not an option everywhere and, in some areas, women had to walk or find alternative means of transportation. This also applied to situations where the ambulances where already occupied and caused first and second delays; and we observed several women arriving at the health facilities only in second-stage labour. Moreover, both ambulance and private transportation was not always reliable since vehicles were usually old and often worn out, thereby risking critical delays as this interviewee explained:

“On the way, the motor broke, and they had to call here – we hurried and organised transport to fetch her [the woman in labour] … but when we arrived, she had already died, it took so long.” (Person in charge of the maternity waiting home, region 3).

In response to restricted access to means of transportation, women often opted for home births, several interviewees further explained. This was aggravated by indirect OOPs for transportation. While PIMI was still financed by the EU, ambulance transportation was free of charge for birthing women during pick-up and referral. With EU financing having ceased, this changed in most places, and women now needed to pay for the gasoline. However, not all could afford this, leading to a decline in peripartum care uptake and delays, as this interviewee explained:

“It’s very difficult nowadays, now, for the ambulance to pick you up, you are charged because the programme has died and you have to cover the gasoline… if you don’t have the means, some risk it and walk and give birth on the way.” (Nurse responsible for MCH services, health centre, region 2).

During observations, we also noted that price negotiations for the ambulance transport regularly caused delays in referrals. Furthermore, the imposition of indirect OOPs for the transportation from the health facility to the regional hospital regularly put women and their companions under severe stress about the financial burden on how to afford the OOPs (about 6,000–8,000 XOF, equivalent to 10–13 USD). To avoid OOPs and risking giving birth on the way, one provider explained that they commonly recommended women to move close to the health facility during the last pregnancy month and await the birth e.g., at a family member’s place. For women with diagnosed high-risk pregnancies, maternity waiting homes provided the opportunity to await the birth close to hospitals in some regions.

### Material constraints compromising uptake, timeliness and quality of care

Both IDIs and observations revealed that peripartum care was provided under severely resource-constrained conditions. Material constraints were ubiquitous and encompassed essential medicines, consumables, and equipment. Interviewees explained:

“[Equipment, medicine, ] all of that is lacking, that’s why I’m saying we don’t have material resources.” (Head nurse, health centre, region 1).

“We are particularly lacking medicines – here, we are lacking more than what is available…we also don’t have a lot of equipment – we have birthing tables and what we need for the registration, but we are lacking a [foetal] doppler and a blood pressure monitor.” (Head midwife, regional hospital, region 2).

Accordingly, during observations, we noted that essential medicines and consumables including uterotonics, anaesthetics, tetanus vaccines, gloves, catheters, reagents for tests, and disinfectants were regularly either scarce or unavailable. In response to shortages of gloves and disinfectants, providers commonly omitted monitoring and documentation tasks that would require sanitising their gloves or putting on new ones, entailing substandard monitoring and documentation. Another tactic was to shift tasks requiring gloves to the staff member wearing them, if possible. This, however, sometimes necessitated alternating between women with barely sanitised gloves, entailing compromised patient safety, delays, and diffusion of responsibilities. Occasionally, examinations and diagnostics were also performed without gloves, thereby compromising both occupational and patient safety. Meanwhile, we regularly observed only superficial cleaning of utilised equipment and surfaces in absence of sufficient disinfectants, resulting in the utilisation of insufficiently cleaned equipment. If materials for a test were unavailable, e.g., reagents, providers usually responded by forgoing performing it. Scarce materials such as uterotonics were regularly rationed to women in greatest need.

IDIs and observations revealed similar practices in response to lacking appropriate equipment. While one provider at a regional hospital explained that they needed to share the maternity ward’s only blood pressure monitor between consultations and childbirth, we observed the same practice at another regional hospital. Here, the only functioning manual blood pressure monitor was shared between the observation, delivery, and operating rooms, thereby limiting regular blood pressure monitoring of the birthing women. During observations, we also frequently noted the use of broken equipment, such as manual blood pressure monitors with broken cuffs, birthing tables lacking leg support, or stretchers falling apart. Furthermore, one provider explained that their hospital’s birthing table capacity was insufficient. In response, women frequently had to give birth on the floor:

“Often somebody ends up pushing the baby out on the floor since both birthing tables are already occupied.” (Head midwife, regional hospital, region 2).

During observations at the health centre, we noted the same practice. Several interviewees also reported a shortage of observation beds at the health facilities alongside several tactics to cope with it: to triage women and children postpartum and discharge those with no or the fewest complications upon full capacity; limiting the postpartum observation time for all; and repurposing hallways or the adjacent maternity waiting home for observations.

Interviewees commonly explained shortages of essential medicines and consumables by PIMI ‘having ended’ 4–5 months prior to the IDIs, when EU funding ceased, which had brought health facility supplies to a halt. In response, providers now prescribed unavailable materials for private purchase at pharmacies, entailing direct OOPs. However, across observations and IDIs, we noted that women were frequently unable to pay, leading to omissions of diagnostics and treatments and third delays. For example, one interviewee explained:

“Yesterday and the day before, I had meetings with the medical doctor because they had prescribed medicine [to a woman in our maternity waiting home] – but she did not buy it because she did not have the money. During one or two days, she did not buy and take the medicine she was supposed to take – and she came here with complications … she also should have done some tests which cost around 10–11,000 [XOF, equivalent to 19–21 USD] – but she did not have the money… when I asked her, she said ‘no, my mother will bring me money tomorrow afternoon’. But when she came, she only brought 4,000 or 6,000 [XOF, equivalent to 8 and 12 USD, respectively], so it was still insufficient.” (Person in charge of the maternity waiting home, region 3).

Another provider explained that they were unable to perform physical examinations when women were unable to pay for gloves. At the same time, during observations, we noted that providers repeatedly blamed women for compromising their ability to provide good care due to their inability to pay for required materials or delays in purchasing them.

During observations, we noted that private material purchases often caused delays since birth companions needed to arrange the money and go to the pharmacy, which was usually also closed at night. To provide for OOPs, interviewees reported that women commonly continued their usual economic activities such as field cultivation or fishing and selling the products on the market during pregnancy, which involved hard physical labour and increased risks of pregnancy complications. Observations also revealed that OOPs were unpredictable, thereby compromising women’s financial preparedness: Firstly, pharmacies were private and prices for prescribed materials changed; secondly, unanticipated complications and interventions, e.g., emergency caesarean sections, entailed unanticipated material demands. At the same time, health workers’ false expectations of the utility of prescribed materials sometimes contributed to higher OOPs. For example, we noted several prescriptions of more expensive sterile gloves reflecting some providers’ conviction that sterile gloves were superior to non-sterile gloves in preventing HIV transmission, despite providing no superior protection.

In response to the various direct and indirect OOPs, many families dismissed facility births as *“only expensive*,* only tiresome”* (head midwife, regional hospital, region 1), and took the decision on whether care seeking was worthwhile based on the woman’s anticipated risk, as this interviewee explained:

“The family makes the decision: [For primipara, ] they usually say ‘let’s go [to the health facility] whatever it costs, we will pay’, which is because it is a risk to stay at home with the first birth…but those grand multipara, they opt for home births – if something goes bad, they will remember to come to the hospital but if not, they stay at home until they come here and ask for a vaccination card for the child.” (Nurse responsible for MCH services, health centre, region 2).

Accordingly, another provider explained, first delays were common, and we observed women arriving at health facilities after having attempted a home birth but experienced complications such as haemorrhage.

### Human-resource constraints compromising timeliness and quality of care

Material constraints were commonly reported to coincide with severe staff shortages, as this interviewee explained:

“Currently, we are serving a population of over 25,000 persons in this health area … we have one medical doctor, six nurses, one midwife, and one auxiliary midwife … we don’t manage to provide all services the way they should be provided, we don’t manage.” (Head nurse, health centre, region 1).

To attend births and emergencies with available staff, the provider further explained, they were prioritised over preventive services such as ANC and childhood vaccinations. Thereby, women waiting for preventive services sometimes needed to return another day if the demand exceeded capacity. Meanwhile, during health centre observations, we noted that providers regularly applied strong manual fundal pressure to expedite the birth, putting women into severe discomfort and pain. This seemed to be a response to both staff shortages and compromised possibilities to react to potential complications associated with prolonged labour in absence of sufficient uterotonics and surgical capacities. Further, we observed that providers commonly kept their communication with the women very short and omitted explanations about performed tests and procedures, including vaginal examinations, and their results. This seemed to be a tactic to save time. In addition, excess demand entailed compromised responsiveness to emergencies, this interviewee explained:

“If you don’t have anyone you can ask for help and it’s only you and the doctor [on shift], imagine, you and the doctor are assisting a birth and then someone has a haemorrhage – and someone else develops convulsions – and you are in the middle of this! Often, we must ask our cleaning staff to help out in such situations, but it’s a risk.” (Head midwife, regional hospital, region 1).

In response to staff shortages, we noted a high involvement of birth companions, i.e., women’s relatives, during observations. Birth companions’ roles were regularly focussed on diverse practical tasks supporting the women during the health facility admission, such as watching over the woman during labour and the woman and child postpartum and calling for help if necessary, cleaning during and after the birth, washing the woman, and handling the child’s body in case of a perinatal death. Meanwhile, women and newborns were often left unattended by health workers during labour and in the postpartum period.

During observations, providers commonly voiced feeling overburdened amid staff and material shortages. In addition, they explained that their salaries were low and erratic, and we observed frequent staff absenteeism. *“The government has forgotten us and does not care about health care*,*”* one provider explained during observations. Disputes about working conditions also led to regular prolonged strikes, as it was the case during our IDIs. Here, a minimum service level granting access to facility births and emergency treatment of pregnancy complications was usually provided, but no ANC. Yet, emergency services were not always available: At one health facility, a recent maternal death during a strike was ascribed to the anaesthetist not agreeing to come in and assist a caesarean section. Hence, after critical delays, one interviewee explained that health staff attempted to refer the woman to the national hospital in Bissau, but she reportedly died on the way.

Meanwhile, we observed suboptimal workflows and several instances of recommended practices being underperformed. For example, during observations, foetal heartbeat and engagement were regularly not monitored intrapartum and no birth was monitored using partograms. This was despite partograms being generally available, and their use being set as one of PIMI’s performance indicators. To comply with PIMI’s reporting requirements, partograms were usually filled out after the birth. Here, midwives also seemed unsure how to fill them out while voicing that they deemed partograms useless. Similarly, APGAR scores were usually estimated retrospectively. At the same time, clinical practice guidelines defining which interventions needed to be performed and documented when and by whom seemed absent, reflected by a randomness of performed interventions. For example, while some birthing women received blood pressure monitoring upon admission and a postnatal check-up before discharge, this was not always the case, even when the necessary materials seemed available. We also noted a general underutilisation of early skin-to-skin contact, which was limited to small babies. Other babies were usually placed on a neonatal table, and regularly left unattended while taking care of the mother and documentation tasks. We also noted that providers usually did not instruct on early breastfeeding. If interventions were performed, they were frequently either not documented or, in the case of diagnostics, only noted on small pieces of papers. Such papers got frequently lost or were confused between patients, which became evident particularly during shift changes, complications, and referrals.

## Discussion

In this study, we found a crucial mismatch between strategies being employed to promote women’s uptake of facility-based peripartum care and the standard of services being provided: On the one hand, health care providers considered health facilities as the only reasonable place of birth and encouraged women to seek facility births. On the other hand, geographical, material and human-resource constraints severely compromised timeliness and quality of provided peripartum services. Providers voiced feeling overburdened amid shortages of appropriate equipment, essential medicines and consumables, and staffing. To navigate this context, they applied various adaptation strategies, such as prescribing materials for private purchase, omitting tests, and delegating tasks to birth companions. These strategies had several consequences, including compromised patient and occupational safety, delays, and diffusion of health worker responsibilities. Suboptimal clinical workflows and an apparent absence of clinical practice guidelines further contributed to substandard care and delays. Meanwhile, ubiquitous and partly unpredictable OOPs, linked to geographical and material constraints, placed a high financial burden on birthing women and their families, and caused delays while further compromising access to both peripartum care and ANC. In response, providers explained that women would condition care seeking on perceived birthing risks: If deemed high-risk, they would prioritise seeking facility-based peripartum care, if not, they would opt for home births.

The challenges identified in this study are by no means unique to Guinea-Bissau. A large body of evidence highlights geographical constraints, including long distances and poor road networks, compromised availability of transportation, and financial concerns as pivotal barriers impacting timely access to essential MCH services across sub-Saharan Africa [40–43]. Similarly, the imposition of user fees despite official user-fee waivers has been reported [44–46]. At the same time, the global shortage of health workers is well acknowledged, with low- and middle-income countries being most affected [47]. Material and human-resource constraints impacting quality and timeliness of MCH services have also previously been highlighted in rural sub-Saharan Africa [48, 49].

Alarmingly, the challenges identified in this study are also largely congruent with barriers to essential MCH services reported both before [50] and earlier during PIMI’s nation-wide implementation [21] in rural Guinea-Bissau. This includes geographical barriers, OOPs [21, 50], and shortages of adequate materials and human resources at health facilities [50]. The persistence of these barriers over time suggests that they were insufficiently addressed by PIMI’s design.

To address geographical barriers, PIMI provided free ambulance pick-up and referral for birthing women. However, as detailed by the health care providers in our study, this measure was inadequate considering the big catchment areas of health facilities along with long distances, poor road conditions, and few available vehicles. Further, financial barriers arose from the discontinuation of related subsidies following the donor transition. To sustainably reduce geographical barriers, improving Guinea-Bissau’s road infrastructure seems key but highly cost- and time consuming. Likely easier realisable in the short term would be an improvement of access to adequate emergency transportation. Here, a multitude of different options have been discussed in low- and middle-income settings, including financing schemes to enable women and communities to self-organise transportation, and ensuring the availability of context-appropriate ambulances, e.g., motorbikes, tricycles, and 4 × 4-wheel ambulances [51]. Future implementation research is required to identify solutions which are adequate for the Bissau-Guinean context, fundable and responsive to the needs of women, communities, and the health system.

To address material constraints, PIMI supplied medical equipment, essential medicines and consumables, and rehabilitated and maintained health facilities while being funded by the EU [17, 19]. Yet, in our study, we found a persistent lack of essential material supplies alongside often worn-out health facility equipment. While lacking supplies of essential medicines and consumables were explained by the cease of EU funding in July 2021, shortly before we conducted the IDIs, the supplies were still discontinued during our observations, 4–5 months later. In absence of adequate domestic resources enabling bridging this gap in donor support, this caused severe quality-of-care concerns alongside financial barriers to care since service providers resorted to prescribing some of the lacking materials for private purchase. Hence, the funding gap conditioned accessibility of care on individual ability to pay. Being aggravated by the observed common unpredictability of the resulting OOPs and indirect OOPs required for transportation, this structurally disadvantages women with low purchasing power and leads to severe equity concerns [52], while compromising the human right to health [53]. Thus, our findings call for the prioritisation of global health funding securing a continuous supply of essential medicines and consumables while underscoring the need for strengthened donor coordination promoting sustainability of supported interventions.

To address human-resource constraints, PIMI provided health worker trainings [17, 19, 54]. Yet, rather than knowledge gaps, providers commonly emphasised staffing shortages as a key concern. The reported health worker density of 9 health professionals for a population of over 25,000, detailed by one provider in our study, corresponds to the country’s previously reported health worker shortage [24] and low physician density (2 per 10,000 people), which ranks among the lowest in the world [25]. It further falls considerably short of the WHO recommendation of at least 23 health care professionals per 10,000 population [55]. Such severe understaffing requires urgent action. Meanwhile, against this background, it needs to be questioned how much impact can be expected from training interventions if not tailored to assist with task prioritisation and performing triage to navigate the situation. At the same time, health workers in our study voiced how they felt overburdened by bearing the responsibility of navigating ubiquitous constraints compromising their ability to provide good care. Similar circumstances have been documented in other rural settings in sub-Saharan Africa [48, 49], and may lead to moral injury, diminished work motivation, and ultimately staff absenteeism in an already understaffed system. Hence, improving local working conditions, including ensuring regular salary payments, appears a necessary step to both disburden the local workforce and provide a fair framework of their employment. Further, opportunities for task shifting between occupational cadres and/or formal health care providers and community health workers could be explored, while prioritising the allocation of global health funding to training and employing more health workers.

Finally, our findings reflect that there are several low-hanging fruits, which seem highly promising in promoting the quality of peripartum care provision. We observed a range of missed clinical opportunities, including a general underutilisation of clinical practice guidelines for routine and emergency peripartum care, and an underperformance of low-resource approaches such as early skin-to-skin contact, breastfeeding, and effective documentation. Similar missed opportunities have been documented in other sub-Saharan African settings, e.g., in rural Burkina Faso [48]. In addition, partograms were regularly documented retrospectively, a practice that has also been reported, e.g., in rural Burkina Faso [56], turning partograms into an ineffective documentation task instead of a purposeful, prospective guidance to quality peripartum care provision. To ensure the provision of timely evidence-based, low-resource interventions while potentially disburdening health care providers by avoiding duplicate work, it seems pressing to address this by developing or updating local clinical practice guidelines. Here, contextualisation and local ownership seem key to warrant the appropriateness and acceptability of the guidelines, which may be achieved by involving local health workers in their definition or revision [57].

### Strengths and limitations

Our study was embedded in a wider research project assessing PIMI’s effects on essential MCH service coverage and perinatal mortality [20] and barriers to care [21]. This allowed us to capitalise on prior insights and provided a robust basis for interpretation. This was further promoted by the contextual understanding and knowledge of Guinea-Bissau Creole of all researchers involved in the data collection, member checking, as well as discussions of preliminary findings with service providers from the observation sites. Yet, while we aimed to include all target health facilities of previously in-depth-interviewed women [21] in this study, one target health centre in region 3 was inaccessible when we conducted the IDIs.

Applying a systems thinking lens aided by theories of social practice allowed us to explore the complex socio-material interactions shaping the provision of timely and quality peripartum care during PIMI in rural Guinea-Bissau. Further, it enabled us to gain an in-depth understanding of the breadth of factors and practices affecting compromised timeliness and quality of services and system adaptations employed by providers and women, some of which appear immediately actionable. This would not have been possible with more positivistic enquiries.

Combining IDIs with participant observations allowed us to compare providers’ stated views with observed practices, thereby promoting internal validity and mitigating social desirability influencing our findings. While our presence in the field may have influenced behaviours of providers, women, and their companions during observations, our field stays were long and continuous over several weeks and we would expect behavioural adaptations to diminish over time. Yet, we found no such changes in behaviours. Furthermore, the circumstance that providers openly showed behaviours deviating from stipulated guidelines, e.g., partograms being filled out retrospectively, suggests that providers did not modify their behaviour based on our presence. In the maternity waiting homes, we interviewed non-medical personnel with limited technical insights into peripartum care provision. However, their involvement provided us with deeper insights into challenges at the two regional hospitals where we did not conduct direct observations. Meanwhile, it allowed for comparisons of findings reported by hospital and maternity waiting home staff, thereby further promoting internal validity.

Being a qualitative study and thereby based on a small sample size, our findings cannot be generalised beyond the study sites. In this context, it is important to note that we restricted our observation sites to two health facilities to allow gaining in-depth knowledge of clinical realities. However, we found many inter-facility similarities suggesting that identified challenges may be similar across rural Guinea-Bissau. Further, the interpretation of findings may have been influenced by the European upbringing and acquaintance with person-centred, resource-rich health systems of some of the research team members. We continuously reflected upon resulting preconceptions during the research process by discussing observations and findings with the Bissau-Guinean team members, other colleagues knowledgeable of the Bissau-Guinean MCH service provision landscape as well as peripartum care providers from the observations sites.

## Conclusions

This study identified geographical, material and human-resource constraints severely compromising the timeliness and quality of peripartum care provision in rural Guinea-Bissau. This was despite the implementation of a large-scale health system strengthening initiative aiming at promoting the access to and quality of MCH services. The diversity of factors compromising the provision of timely and quality peripartum care highlights the need to carefully consider the full picture of systems constraints and adequate solutions to address them in the design of health systems strengthening initiatives. This appears particularly important to avoid promoting demand in a system which is unfit to adequately respond. Meanwhile, our findings underscore the importance of local leadership in the design of health systems strengthening interventions to enable the effective identification of systems constraints and adequate solutions to address them. Furthermore, unfavourable systems adaptations in response to persisting geographical, material and human-resource constraints highlight the need for continuous monitoring of the implementation and effects of health system strengthening initiatives, particularly during programme changes such as donor transitions.

### Electronic supplementary material

Below is the link to the electronic supplementary material.

Supplementary Material 1

## Data Availability

Data from this study contains identifiable information of sensitive nature related to peripartum provider-mother interactions, personal and health facility characteristics which can only partially be anonymised. Therefore, we assured our study participants that we would treat their information confidentially and refrain from sharing raw data. For further information, please contact SM Damerow, sdamerow@health.sdu.dk.

## Declarations

### Reflexivity statement

The authorship team includes six female and one male author with levels of seniority ranging from early career scholar to full professor. Two authors are Bissau-Guinean, five European, of whom three have been working in Guinea-Bissau. All authors have extensive experience from living and conducting research in low- and middle-income settings. The authors’ academic backgrounds are diverse and include public health, global health, psychology, nursing, and medicine including gynaecology and obstetrics.
