# Supplementary material for: Timeliness and quality of peripartum care provision during a health system strengthening initiative in rural Guinea-Bissau: a qualitative situation analysis
Source: BMC Pregnancy Childbirth. 2024 Jul 13;24:478. doi: 10.1186/s12884-024-06669-8 (PMC11245816; doi:10.1186/s12884-024-06669-8)
Supplement: Supplementary file 1 — Supplementary Material 1 [file 12884_2024_6669_MOESM1_ESM.docx]

**Timeliness and quality of peripartum care provision during a health system strengthening initiative in rural Guinea-Bissau: A qualitative situation analysis**

*Supplementary material*

[1. Interview guide 2](#_Toc169688184)

[2. Observation guide 6](#_Toc169688185)

# Interview guide

***The following interview guide is a translation of the original guide developed in Portuguese.***

**General information**

| Date |  |
| --- | --- |
| Start / end time |  |
| Name of interviewer |  |
| Name of other investigators present |  |
| Region |  |
| Name of health facility |  |
| Name of informant |  |
| Employment role |  |

**Section 1: Introduction**

Thank you for taking the time to have this interview with us today. Our names are _________________ and we are research assistants at the Bandim Health Project.

We are here to learn from you about care seeking choices during childbirth and why women choose one place over another. Therefore, we would like to ask you some questions and hear your opinions. When we ask you a question you are welcome to tell us what you think. There are no wrong or right answers, and you can say anything you like. So please feel free to comment on anything. We are here to learn from you.

Participation is voluntary and if there are any questions you don't want to answer, you don't have to, just let us know. You can also ask any questions you may have. This interview will last approximately one hour. Would you like to participate in this interview? *[If yes: fill in the consent form]*

As it can be difficult to take notes fast enough, we would like to record this interview. Then we can go home and listen to the recording and better understand and remember what you said. Your responses are confidential so we will not mention your name and the recording will be used for the purpose of research only. Is it ok that we record the interview? *[If yes:* *switch on recording device]*

Do you have any questions before starting?

**Section 2: Work profile in relation to MCH care**

Now we would like to learn what kind of services you provide at this health facility - and your specific role.

| **Main questions** | **Probes** |
| --- | --- |
| Can you explain to us what kind of MCH services are provided at this health facility?  How many births happen here each day/week/month?  Can you describe your role in relation to the services that are provided at this health facility? | *Clarify details.*  *Clarify whether emergency obstetric care services are provided (caesarean section / other services) and what happens in the event of an emergency.*  *Clarify whether women can be admitted.*  *Ask about the most appropriate time frame, e.g. hospital births: day*  *Clarify details, e.g.:*   - Can you describe your usual working day? - What proportion of your work is in the field of MCH? - How long have you worked in [this position]? - Do you have any special training? - Do you have colleagues performing similar tasks who can support or substitute for you if you are, for example, ill? |

**Section 3: Choices of care for MCH services**

Now we would like to learn what kind of MCH services women in this region usually use.

| **Main questions** | **Probes** |
| --- | --- |
| Can you help us reconstruct/understand a pregnant woman's journey through formal and informal MCH services, from the moment she is planning/noticing a pregnancy to the birth of her child?  What do you think are the most important factors that determine the care-seeking choices?  Do you think the journey you describe is ideal? | *Clarify details, e.g.:*   - Which formal and informal service providers would a woman normally use? In which sequence? - Where are the providers located? Are they near the villages? - In which cases would women opt for MCH service providers in the formal public health system? - When would they choose to use traditional healers or traditional midwives?   *Clarify details, e.g.:*   - Are there women who would decide differently from the scenario you described? What could characterise them [ethnicity / parity / place of residence / socio-economic status / ...]? - Which characteristics do you think are decisive? Why? - Do you notice differences in care-seeking behaviour between routine and emergency services? What do you think determines these differences? - Do you think care-seeking behaviour has changed over time? Why do you think this is?   *Clarify details, e.g.:*   - What should be different? |

**Section 4: Characteristics of this service provider**

Now we would like to learn more about your health facility.

| **Main questions** | **Probes** |
| --- | --- |
| How do women usually get here?  How long do you think they usually have to travel to get here? | *Clarify details, e.g.:*   - Does this change in cases of emergency? - Is there any emergency transport available for patients to get here? Which kind (ambulance)? - How much do women usually pay to be picked up by an ambulance? |
| Can women always come and give birth here - or only at scheduled times?  Do you think you have enough capacity to provide all the services that women and their children need?  How long do women who come here to give birth usually have to wait for attendance?  How many women does a team member usually help at the same time? | *Clarify details, e.g.:*   - Do you / does this health facility open every day? - Are the health professionals here every day? All day / when? - Are there days / occasions [e.g., staff training] when this is different? - How do patients know about changes in opening hours? - Are there differences between routine and emergency services?   *Clarify details, e.g.:*   - What should be different? Why? - Do you have enough staff to attend to all the women and children who come here? Always / usually / are there situations where this is different? - Do the staff who work here have the appropriate educational background / skill set for the tasks? - Do you have all the equipment and medicines available to do your job for pregnancy consultations and deliveries?   *Clarify details, e.g.:*   - Are there situations in which this is different (e.g., emergencies)? - What do patients usually do while they wait?   *Clarify details.* |
| When a woman comes here to give birth, what does she need to bring?  Do you provide food here or does the family usually bring food?  How much do women usually pay here for births (normal / stitches / caesarean section, if applicable)?  How do women usually arrange the money? | *Clarify details.*  *Clarify details.*  *Clarify details, e.g.:*   - For what kind of materials/medicines? - Do women need to pay for the bed (“hotel costs”)? - Are there any diagnostics/analyses women need to pay for?   *Clarify details, e.g.:*   - Do you notice that women sometimes have difficulties paying? - What happens if they have difficulties paying? |
| Are you aware of any concerns or problems that prevent women from seeking care here (childbirth) or that need to be overcome in order to come here? | *Clarify details including consequences, e.g.:*   - What concerns / problems? - Are you aware of the actions women are taking to overcome them? What kind of actions do they take? |

**Section 5: Decision maker for seeking MCH care (childbirth)**

| **Main questions** | **Probes** |
| --- | --- |
| In your experience, who decides when and where to seek care for childbirth? | *Clarify details, e.g.:*   - Do you think women usually discuss their choices with someone? With whom? Do you think this influences the final decision of when and where to seek care? - Do you think this differs in emergency situations? How does it differ? Why? |

**Section 6: Closing questions**

| **Main questions** | **Probes** |
| --- | --- |
| In this area, there is a [high/low] coverage rate of facility births. Why do you think this is the case? | *Clarify details, e.g.:*   - What's being done differently here compared to other health areas? |
| What do you think is the biggest challenge that women have to overcome in order to give birth here? | *Clarify details and ask for examples.* |
| If you had the power to change things, what would you do to improve maternal and child health in this area? | *Clarify details and ask for examples.* |

**Section 7: Member checking and closing**

Thank you very much for sharing your knowledge and experiences with us. I have no further questions. Do you have any questions about our research or final comments to this interview?

We would now like to make some notes about what we talked about to make sure we haven't forgotten anything. Would it be ok if we came back later for a short chat to see if you had any more questions and make sure we understood everything?

*Reflect and discuss and summarise the main points about barriers and facilitators mentioned in the interview.*

*After discussing and summarising the main points:* The impression we got from our conversation was that *[summarise the key points mentioned in the interview].*

• Is that correct? Is there anything else you'd like to add?

Thank you very much. This is very valuable information for us, so thank you very much for your participation.

# Observation guide

| **Focus areas^1^** | **Material factors** | **Competence factors** | **Symbolic factors** | **Relational factors** | **Motivational factors** |
| --- | --- | --- | --- | --- | --- |
| **Waiting area**  *General observations:*  *- Who is present? How many patients are waiting?*  *- Who is attending the waiting area? Visible service providers?*  *- How is the waiting time spent?* | -Time spent waiting  - Means of transportation to the health facility  - Payments or other means to secure access to services/referrals | - Triage, structure, and registration of the waiting women  - Divisions of tasks, patients | - Communication between women and service providers  - (Dis-)respectful behaviour, stigma | - Power dynamics between providers, patients, and relatives | - Professional ethics  - Performance indicators (incl. changes associated with PIMI) |
| **Antenatal care**  *General observations:*  *- How is the consultation room/area organized?*  *- Who is attending the consultation?* | - Adequacy of physical environment for antenatal care  - Availability of adequate equipment, tests, medicine, guidelines for examination and care  - Availability of clean water, soap/disinfectants, electricity  - Payments or other means to secure adequate materials/access to services/referrals | - Information provided  - Health education provided  - Maternal and foetal assessment performed  - Preventive measures performed  - Registration of data  - Cleanliness | - Communication between women and service providers  - (Dis-)respectful behaviour, stigma  - Privacy and confidentiality | - Power dynamics between providers, patients, and relatives  - Collaboration  - Decision-making (incl. leadership and hierarchies)  - Patient/relative involvement | - Professional ethics  - Performance indicators (incl. changes associated with PIMI) |
| **Intrapartum care**  *General observations:*  *- How is the delivery room/area organized?*  *- Who is present during the birth?*  *- 'Traffic' during labour and birth (who is coming and going)?* | - Adequacy of physical environment for intrapartum care  - Availability of adequate equipment, tests, medicine, guidelines for examination and care  - Availability of clean water, soap/disinfectants, electricity  - Referral and emergency obstetric care opportunities  - Payments or other means to secure adequate materials/access to services/referrals | - Information provided - Registration of data - Maternal and foetal assessment performed (incl. partograph use) - Routine care and support provided (e.g., pain relief, urination, ambulation, eating/drinking)  - Medicine administration  - Resuscitation processes - Cleanliness | - Communication between women and service providers  - (Dis-)respectful behaviour, stigma  - Privacy and confidentiality | - Power dynamics between providers, patients, and relatives  - Collaboration  - Decision-making (incl. leadership and hierarchies)  - Patient/relative involvement | - Professional ethics  - Performance indicators (incl. changes associated with PIMI) |
| **Postnatal care**  *General observations:*  *- How is the post delivery room/area organized?  - Who is present?*  *- 'Traffic' (who is coming and going)?* | - Adequacy of physical environment for postnatal care  - Availability of adequate equipment, tests, medicine, guidelines for examination and care  - Availability of clean water, soap/disinfectants, electricity  - Referral and emergency obstetric opportunities  - Payments or other means to secure adequate materials/access to services/referrals | - Information provided  - Registration of data - Routine postpartum maternal and neonatal assessment and care provided (incl. skin-to-skin contact and breastfeeding initiation) | - Communication between women and service providers  - (Dis-)respectful behaviour, stigma  - Privacy and confidentiality | - Power dynamics between providers, patients, and relatives  - Collaboration  - Decision-making (incl. leadership and hierarchies)  - Patient/relative involvement | - Professional ethics  - Performance indicators (incl. changes associated with PIMI) |

^1^ In addition to the listed categories, we also noted when other life practices (e.g., family, work, and other obligations) appeared to interact with the focus areas.
